# Increased life span from overexpression of superoxide dismutase in *Caenorhabditis elegans* is not caused by decreased oxidative damage

**DOI:** 10.1016/j.freeradbiomed.2011.07.020

**Published:** 2011-10-15

**Authors:** Filipe Cabreiro, Daniel Ackerman, Ryan Doonan, Caroline Araiz, Patricia Back, Diana Papp, Bart P. Braeckman, David Gems

**Affiliations:** a Institute of Healthy Ageing and Research Department of Genetics, Evolution and Environment, University College London, London WC1E 6BT, UK; b Laboratory for Aging Physiology and Molecular Evolution, Department of Biology, Ghent University, Ghent, Belgium; c Department of Medical Chemistry, Semmelweis University, 1094 Budapest, Hungary

**Keywords:** HNE, 4-hydroxynonenal, AMPK, AMP-dependent kinase, CML, carboxymethyllysine, co-OE, co-overexpression, HSF-1, heat shock factor-1, IIS, insulin/IGF-1 signaling, NAC, *N*-acetylcysteine, OE, overexpression, ROS, reactive oxygen species, RNAi, RNA-mediated interference, O_2_^•−^, superoxide anion, SOD, superoxide dismutase, Aging, *Caenorhabditis elegans*, *daf-16*/FoxO, ER stress, Oxidative damage, Superoxide dismutase, Free radicals

## Abstract

The superoxide free radical (O_2_^•−^) has been viewed as a likely major contributor to aging. If this is correct, then superoxide dismutase (SOD), which removes O_2_^•−^, should contribute to longevity assurance. In *Caenorhabditis elegans,* overexpression (OE) of the major cytosolic Cu/Zn-SOD, *sod-1,* increases life span. But is this increase caused by enhanced antioxidant defense? *sod-1* OE did not reduce measures of lipid oxidation or glycation and actually increased levels of protein oxidation*.* The effect of *sod-1* OE on life span was dependent on the DAF-16/FoxO transcription factor (TF) and, partially, on the heat shock TF HSF-1. Similarly, overexpression of *sod-2* (major mitochondrial Mn-SOD) resulted in life-span extension that was *daf-16* dependent. *sod-1* OE increased steady-state hydrogen peroxide (H_2_O_2_) levels in vivo. However, co-overexpression of catalase did not suppress the life-span extension, arguing against H_2_O_2_ as a cause of longevity. *sod-1* OE increased *hsp-4* expression, suggesting increased endoplasmic reticulum (ER) stress. Moreover, longevity was partially suppressed by inactivation of *ire-1* and *xbp-1,* mediators of the ER stress response. This suggests that high levels of SOD-1 protein may challenge protein-folding homeostasis, triggering a *daf-16-* and *hsf-1-*dependent stress response that extends life span. These findings imply that SOD overexpression increases *C. elegans* life span, not by removal of O_2_^•−^, but instead by activating longevity-promoting transcription factors.

## Highlights

► *sod-1* over-expression increases levels of cellular ROS and of molecular damage. ► *sod-1* over-expression effects on lifespan are dependent on DAF-16 (FoxO) and HSF-1. ► Increased lifespan is partially suppressed by inactivation of ER stress mediators. ► *sod-2* (MnSOD) over-expression effects on lifespan are dependent on DAF-16.

The oxidative damage theory proposes that accumulation of molecular damage caused by reactive oxygen species (ROS), particularly superoxide (O_2_^•−^) and its derivatives, is a primary cause of aging [1], [2]. The enzyme superoxide dismutase (SOD) converts O_2_^•−^ into hydrogen peroxide (H_2_O_2_), which in turn is converted into H_2_O and O_2_ by the action of catalase, glutathione peroxidase, and other enzymes. The oxidative damage theory suggests that SOD should protect against aging as well as oxidative damage. However, recent studies in *Caenorhabditis elegans* have found little support for this prediction [3], [4], [5]. For example, administration of SOD mimetic compounds fails to increase life span [5], [6], [7] and low concentrations of O_2_^•−^ generators actually increase life span [8], [9], [10]. Moreover, for four of the five *C. elegans sod* genes, abrogation of function has little or no effect on aging [11], [12], [13], [14].

However, the effects of manipulation of expression of *sod-1,* the major cytosolic Cu/Zn-SOD isoform, could imply that cytosolic O_2_^•−^ and the damage that it causes contribute to *C. elegans* aging. Abrogation of *sod-1* by gene deletion or RNA-mediated interference (RNAi) slightly reduces life span [11], [13], [14], [15] and *sod-1* overexpression (OE) can increase life span [11]. However, in that study we did not verify that *sod-1* OE reduces levels of oxidative damage. Moreover, *sod-1* OE lines were hypersensitive rather than resistant to oxidative stress, hinting that SOD OE might not reduce ROS-induced damage in these strains. It is also not known whether SOD levels in wild-type worms are limiting with respected to ROS detoxification.

The effect on life span of transgene-induced elevation of SOD has been tested in several model organisms, with mixed results (reviewed by [16]). Like its effect in *C. elegans,* SOD OE can extend life span in *Drosophila melanogaster*[17], [18], but in mice, ubiquitous overexpression of SOD does not extend life span [19]. A potential problem with this approach to testing the oxidative damage theory is that SOD OE can have a pro-oxidant effect, leading to more rather than less oxidative damage [20], [21]. For example, elevated levels of SOD can markedly increase H_2_O_2_ levels in vivo [22]. There are other instances of manipulations of antioxidant defenses having unpredictable effects on redox status and damage levels. In worms, loss of the peroxiredoxin *prdx-2* leads to up-regulation of genes involved in phase II detoxification [23]. In mice, loss of glutathione peroxidase can lead to health benefits through altered insulin signaling [24]. In humans, the beneficial effects of physical exercise can be blocked by consumption of antioxidants, implying that ROS have a positive role in conferring these health benefits [25].

All this raises doubts as to whether overexpression of *sod-1* in *C. elegans* extends life span by reducing levels of oxidative damage. Given that SOD OE can increase levels of H_2_O_2_ and of damage in some contexts, one possibility is that this occurs in worms and increases life span via hormesis. In this phenomenon, exposure to sublethal stress induces an adaptive response resulting in beneficial effects that include increased life span [26]. In *C. elegans,* life span can be increased by transient exposure to stressors, such as heat and hyperoxia, or chronic exposure to low levels of chemical ROS generators [8], [10], [27], [28], [29]. Hormetic effects on life span can require the FoxO transcription factor DAF-16 [8], [30], which is also required for life-span extension by reduced insulin/IGF-1 signaling (IIS) [31]. This suggests that activation of DAF-16 can mediate hormetic effects, perhaps via mechanisms similar to those operative in long-lived IIS mutants.

If *sod-1* OE extends life span by reducing ROS levels, one would expect to see an associated reduction in levels of oxidative damage. However, if a hormetic effect were occurring, then one would not necessarily expect to see reduced oxidative damage. Moreover, the effect on longevity might require mediators of the stress response, such as DAF-16. In this study we investigate these possibilities. Our results support the second scenario and strongly imply that the longevity of *sod-1* OE lines is not attributable to enhanced antioxidant defense. This means that the longevity of *sod-1* OE lines is not evidence of the veracity of the oxidative damage theory of aging.

## Materials and methods

### Strains and culture conditions

Nematode strains used in this study included the following: DR1563, *daf-2(e1370);* GA114, *daf-16(mgDf50); daf-2(e1370);* GA184, *sod-2(gk257);* GA222, *wuEx118* [*sod-2* gDNA *rol-6(su1006)*]; GA226, *wuEx122* [*sod-1* gDNA *rol-6(su1006)*]; GA228, *wuEx123* [*sod-1* gDNA *rol-6(su1006)*]; GA230, *wuEx125* [*sod-1* gDNA *rol-6(su1006)*]; GA644, *wuEx123* [*sod-1* gDNA *rol-6(su1006)*]; *daf-16(mgDf50);* GA646, *wuIs152* [*sod-1* gDNA *rol-6(su1006)*] *hsf-1(sy441);* GA648, *wuIs152* [*sod-1 gDNA rol-6(su1006)*] *aak-2(ok524);* GA697, *zcIs4* [*hsp-4::gfp*] *wuEx196* [*rol-6(su1006)*]; GA800, *wuIs151* [*ctl-1, -2, -3 gDNA myo-2::gfp*]; GA801, w*uIs152* [*sod-1 gDNA rol-6(su1006)*]; GA804, *wuIs155* [*sod-2* gDNA *rol-6(su1006)*]; GA805, *wuIs156* [*sod-2* gDNA *rol-6(su1006)*]; GA808, *wuIs151* [*ctl-1, -2, -3 gDNA myo-2::gfp*]; *wuIs156* [*sod-2* gDNA, *rol-6(su1006)*]; GA809, *wuIs151* [*ctl-1, -2, -3 gDNA, myo-2::gfp*]; *wuIs152* [*sod-1* gDNA *rol-6(su1006)*]; GA812, *wuIs156* [*sod-2* gDNA *rol-6(su1006)*]; *daf-16(mgDf50);* GA824, *wuEx196* [*rol-6(su1006)*]; GA1001, *aak-2(ok524)* (derived by outcrossing RB754); GA1407, *zcIs4* [*hsp-4::gfp*] w*uIs152* [*sod-1 gDNA rol-6(su1006)*]; GA1423, *zcIs13* [*hsp-6::gfp*] w*uIs152* [*sod-1 gDNA rol-6(su1006)*]; GA1424, *zcIs13* [*hsp-6::gfp*] w*uIs152* [*sod-1 gDNA rol-6(su1006)*]; GA1425, *zcIs13* [*hsp-6::gfp*] w*uIs152* [*sod-1 gDNA rol-6(su1006)*]; GA1429, *zcIs13* [*hsp-6::gfp*] *wuEx196* [*rol-6(su1006)*]; GA1430, *zcIs13* [*hsp-6::gfp*] *wuEx196* [*rol-6(su1006)*]; GR1307, *daf-16(mgDf50)*; and PS3551, *hsf-1(sy441)*. Standard nematode culture conditions were used [32].

### Nematode strain construction

To prepare *C. elegans* lines overexpressing *sod-2,* a genomic DNA fragment containing the *sod-2* coding sequence was amplified by PCR. This was injected into worms at 55 ng/μl, creating a line containing the *wuEx118* transgene array. Primers used for PCR were 5′-TGAATCCTACGGAAAGTGCC-3′ and 5′-TCAATGAATGGACAGGTTTCCC-3′. *wuIs155* and *wuIs156* were created by integration of *wuEx118* using X-irradiation. Integrant lines were outcrossed to N2 six times. Co-overexpression with catalase was achieved by combining the transgene arrays *wuIs155* and *wuIs151* [*ctl-1,-2,-3* overexpression] in a single line. The construction of *wuIs151* and of *wuIs152* [*sod-1* overexpression] has been described previously [11].

### Survival analysis

*C. elegans* hermaphrodites were grown at 20 °C and transferred to plates containing 10 μM fluorodeoxyuridine at the L4 stage of development. Death was scored as described previously [33]. Survivorship of populations was compared statistically using the log-rank and Wilcoxon tests, performed using the application JMP 7.0.1 (SAS).

### N-acetylcysteine (NAC) treatment

Worms were transferred to control or NAC plates as L4/young adults and were transferred to freshly prepared plates every 7 days. NAC was added to plates topically from a freshly prepared NAC stock solution.

### Use of HyPer fluorescent probe to measure in vivo H_2_O_2_ levels in C. elegans

HyPer is a genetically encoded H_2_O_2_-specific fluorescent probe [34]. This yellow fluorescent protein-based probe is both sensitive and specific and the fluorescence measurement is ratiometric and therefore not dependent upon the expression level. *unc-119* mutant worms were transformed by biolistic transformation with a vector containing the wild-type *unc-119* gene, the promoter of the constitutive and ubiquitously expressed *rpl-17* gene, and the *hyper* coding region. Homozygous *hyper* transgenic worms were backcrossed twice into wild-type (N2). *wuIs152* [*sod-1*] was then crossed into the *hyper* strain. Nematodes were cultured on NA agar seeded with *Escherichia coli* K12 as described previously [32]. Transgenic worms expressing *hyper* only (controls) or *hyper* plus *sod-1* OE [*wuIs152*] were raised at 24 °C and harvested 1 day after the L3/L4 molt. Worms were washed with S-buffer (43.55 mM KH_2_PO_4_, 6.45 mM K_2_HPO_4_, and 100 mM NaCl in distilled water, pH 6) and next with 2.5 mM EDTA in S-buffer (to eliminate bacteria adhering to the cuticle) and pipetted as a dense pellet of at least 1000 worms in a black, flat-bottomed 96-microtiter plate well (Greiner). The fluorescent signal was measured over a 10-min period using a Victor^2^ 1420 multilabel counter (PerkinElmer) at 25 °C with the excitation filters FP490 and FP405 and emission filter F535. The data shown represent the average ratios over 10 min of four biological replicates (at least three technical replicates each). A fuller description of the validation of this technique as a measure for standing H_2_O_2_ levels will be published soon by P. Back et al.

### Harvesting nematodes for biochemical assays

Synchronous cultures were initiated via alkaline hypochlorite lysis of gravid adults. Eggs were allowed to hatch overnight in M9 buffer at 20 °C and resulting L1 larvae were grown on NGM plates seeded with *E. coli* OP50. At harvest, late L4 larvae and young adult worms were rinsed off the plates, washed with M9 buffer, and stored at − 75 °C until use. For all assays, three to five replicate worm cultures were used.

### Preparation of protein extracts via sonication

Worm samples were homogenized using a Bioruptor (Cosmo Bio Co., Ltd, Tokyo, Japan) in 2-ml microcentrifuge tubes containing equal amounts of suspended worms and CelLytic (Sigma) and 1× protease inhibitors (Roche). The resulting homogenate was centrifuged at 20,000 rpm for 30 min at 4 °C. The supernatant was collected, and cellular debris and intact worms were discarded. For phosphorylation assays, Phosphosafe buffer (Novagen) was used instead of CelLytic. Protein concentration of the supernatant was determined by the Bradford method (Bio-Rad).

### Immunoblot and molecular damage analysis

Thirty micrograms of each total protein extract was separated by SDS–PAGE and then electrotransferred onto a Hybond nitrocellulose membrane (GE Healthcare Europe GmbH). Western blotting experiments were performed with anti-actin monoclonal antibodies (Santa Cruz Biotechnology) at a 1/1000 dilution, anti-Cu/Zn-SOD polyclonal antibody (USBiological) at a 1/2000 dilution, anti-Mn-SOD polyclonal (USBiological) at a 1/2000 dilution, anti-phospho-AMP-dependent protein kinase (AMPK) antibody (Cell Signaling Technology) at a 1/1000 dilution, and anti-AGE monoclonal antibody (clone 6D12) (Cosmo Bio Co., Ltd) at a 1/1000 dilution. Detection of carbonyl groups was performed with the OxyBlot oxidized protein detection kit (Chemicon International) according to the manufacturer's protocol. Samples (15 μg) of total protein extract were incubated for 15 min at room temperature with 2,4-dinitrophenylhydrazine to form the carbonyl derivative dinitrophenylhydrazone before SDS–PAGE separation. After transfer onto nitrocellulose, modified proteins were revealed by anti-dinitrophenol antibodies. Blots were developed with chemiluminescence using the SuperSignal West Pico chemiluminescent substrate (Perbio Sciences). Films were scanned and the amount of signal of each band or total lane (for carbonyl and carboxymethyllysine (CML)) was quantified by densitometric analysis using ImageQuant TL (GE Healthcare Europe GmbH).

### SOD activity assays

SOD activity was measured using the Oxyselect superoxide dismutase activity assay (Cell Biolabs, San Diego, CA, USA) involving the inhibition of superoxide-induced chromogen chemiluminescence by SOD, according to the manufacturer's instructions. Total protein extracts (150 μg) were used for each measurement. For the measurement of Mn-SOD activity, protein extracts were pretreated with 5 mM NaCN to inhibit Cu/Zn-SOD activity. The absorbance of each well was read using a microplate reader (Infinite 200; Tecan) using 490 nm as the primary wavelength.

### Statistical analysis

All results are expressed as the mean ± standard error of the mean. In cases in which several treatments or genotypes were compared to a single control, ANOVA was used for statistical analysis. In all other cases, we performed paired or unpaired *t* tests depending on the experimental circumstances.

## Results

### sod-1 OE increases levels of protein oxidation

**Fig. 1:**
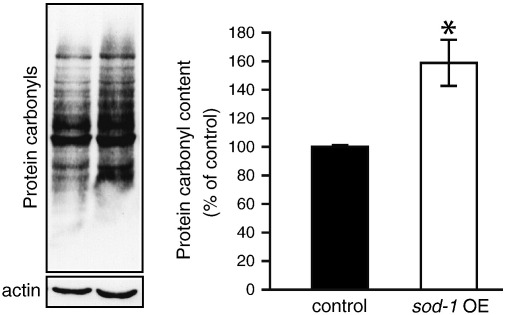
*sod-1* OE does not decrease molecular damage but instead increases protein oxidation. Left: representative OxyBlot showing change in oxidized protein content. Right: quantified changes in protein carbonyl levels. The bar graph depicts means (*n* = 3) for *sod-1* OE ± SE. *P < 0.05 vs control (*rol-6*) (Student's *t* test).

We first tested the effect of *sod-1* OE on three markers of molecular damage: protein carbonyl levels as a measure of irreversible protein damage [13], [14], 4-hydroxynonenal (HNE) protein adduct levels as a measure of lipid oxidation [35], and CML levels as a measure of glycation [36]. *sod-1* OE caused an ~ 1.5-fold increase in protein damage (Fig. 1) and did not increase the levels of HNE or CML (Supplementary Figs. S1A and S1B). This suggests that *sod-1* OE does not decrease levels of molecular damage but, depending on the type of damage measured, either has little effect or even increases it.

Elevated levels of protein carbonyls could be caused by an increase in protein oxidation or by a decrease in the turnover rate for oxidized proteins. To test the latter we measured the activity of the core (20S) proteasome, which is responsible for the degradation of oxidized proteins [37]. No difference was detected in the *sod-1* OE strain (Supplementary Fig. S2). The increased levels of protein oxidation might therefore reflect increased ROS levels.

### Effects of overexpression of sod-1 are daf-16 dependent

The increased levels of protein oxidation, taken together with the reduced resistance to oxidative stress [11], suggest that *sod-1* OE does not increase life span by reducing ROS-mediated damage. An alternative possibility is that *sod-1* OE triggers a protective, hormetic effect that increases life span. To explore this, we tested the dependence of the effects of *sod-1* overexpression on three factors that can mediate responses to stress: AMPK and the transcription factors HSF-1 (heat shock factor) and DAF-16 (FoxO).

**Fig. 2:**
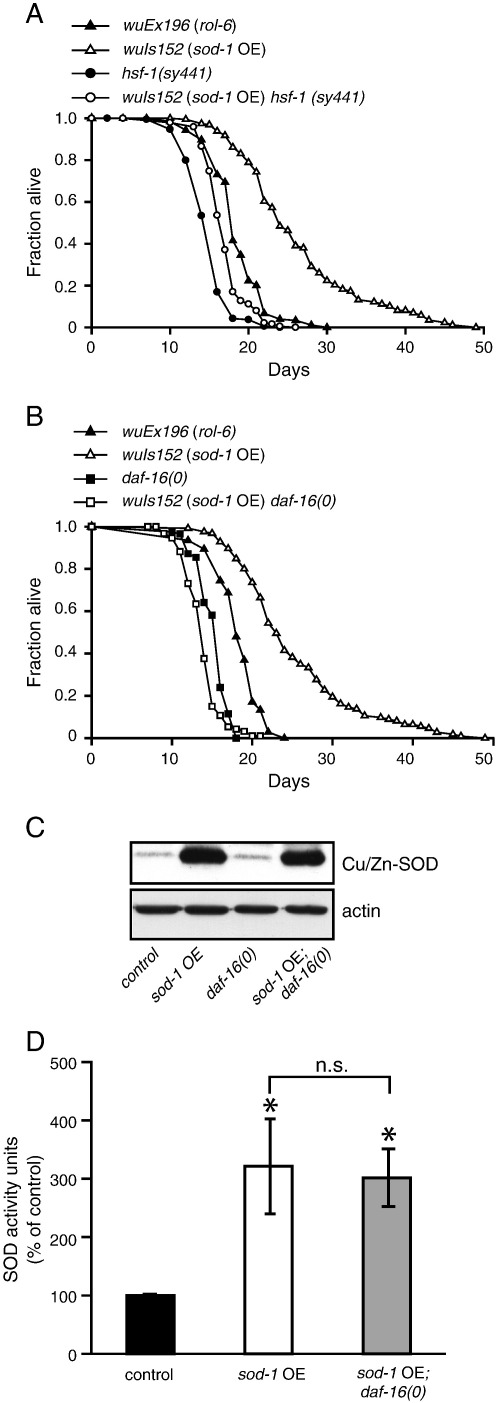
Evidence that life extension by *sod-1* OE is not a simple consequence of enhanced antioxidant defense. (A) *sod-1* OE longevity is partially dependent on *hsf-1*. (B) *sod-1* OE longevity is fully dependent on *daf-16*. (C) *daf-16(0)* does not suppress overexpression of *sod-1*. Immunoblots show the relative protein levels of SOD-1 (~ 18 kDa) in control (*rol-6*), *sod-1* OE line bearing w*uIs152, daf-16(0)* mutants, and *sod-1* OE in a *daf-16(0)* background (*sod-1* OE; *daf-16(0)*). Immunodetection of actin was used as loading control. (D) Total SOD activity resulting from *sod-1* OE. *sod-1* OE results in a threefold increase in total SOD activity compared to the control (*rol-6*). *P < 0.05 vs control (*rol-6*). There was no statistically significant difference between *sod-1* OE (w*uIs152*) lines with or without *daf-16*. Data in bar graphs represent means ± SE. A and B depict representative trials; for full life-span data, including statistics, means, and median values, see Supplementary Table S1.

*sod-1* OE robustly increased the life span of *aak-2* mutants, which lack the major AMPK α subunit (Supplementary Fig. S3 and Table S1), and did not detectably increase AAK-2 phosphorylation levels (Supplementary Fig. S3). This argues against a role for AMPK in the effect of *sod-1* OE on life span. By contrast, the magnitude of *sod-1* OE life-span extension was markedly reduced in an *hsf-1(sy441)* background (a 9% increase in median life span, compared to 33% in *hsf-1*(+)) (Fig. 2A, Supplementary Table S1), suggesting partial dependence on *hsf-1*.

More strikingly, the null mutation *daf-16(mgDf50)* fully suppressed the longevity resulting from *sod-1* OE (Fig. 2B; Supplementary Table S1). Expression of *sod-1* is activated by DAF-16 [11], raising the possibility that the *daf-16* dependence of longevity in *sod-1* transgenic lines reflects effects on *sod-1* expression. However, *daf-16(0)* did not reduce either SOD-1 protein levels (Fig. 2C, Supplementary Fig. S4) or Cu/Zn-SOD activity in *sod-1* OE lines (Fig. 2D). These results suggest that *sod-1* OE extends life span either by activating DAF-16 or by acting in parallel to DAF-16. In addition, we noted that the increase in protein oxidation caused by *sod-1* OE is also *daf-16* dependent (Supplementary Fig. S5)*.* The possible significance of this unexpected observation is considered in the Discussion.

### Overexpression of mitochondrial Mn-SOD (SOD-2) increases life span in a daf-16-dependent manner

The oxidative damage theory views O_2_^•−^ derived from mitochondrial respiration as a key contributor to aging. We therefore tested the effect on life span of overexpression of the major mitochondrial Mn-SOD isoform, *sod-2*[11], [14]. We generated three *C. elegans* lines overexpressing *sod-2* from transgene arrays, *wuEx118, wuIs155,* and *wuIs156. wuIs155* and *wuIs156* are integrated transgene arrays derived from *wuEx118.*

**Fig. 3:**
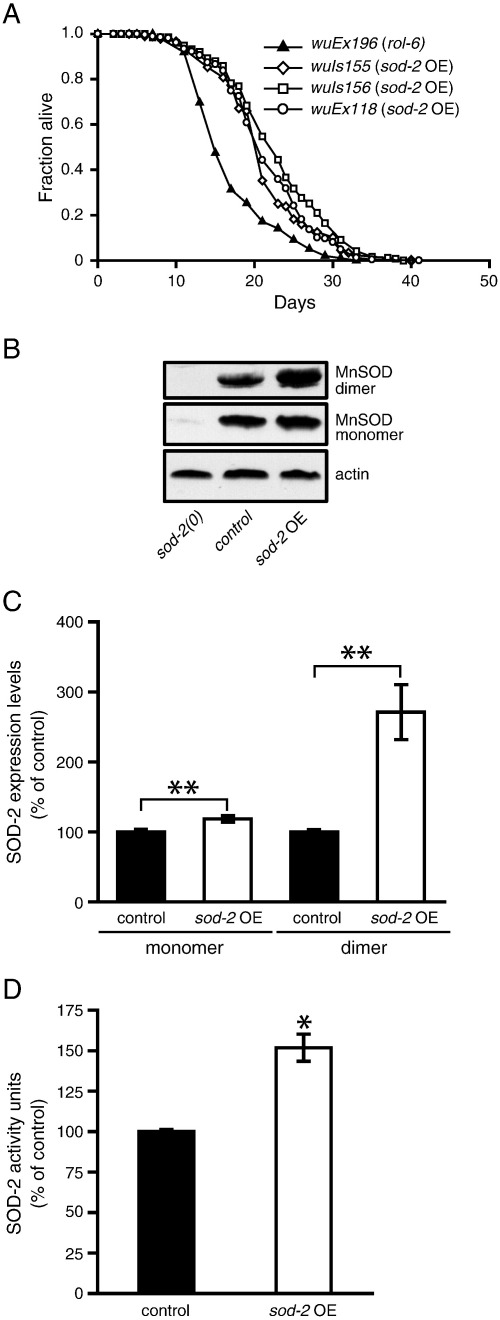
Mn-SOD overexpression reliably increases *C. elegans* life span. (A) Life-span curves with three independent *sod-2* OE lines. A representative trial is depicted; for full life-span data, including statistics, means, and median values, see Supplementary Table S2. (B) Representative Western blot for Mn-SOD in *sod-2* OE line bearing *wuIs156.* Mn-SOD in *C. elegans* functions as a tetramer [52]. Under standard denaturing conditions (heat, SDS, and β-ME), Western blots revealed two bands consistent with the molecular weight of Mn-SOD in dimeric (~ 46 kDa) and monomeric (~ 23 kDa) forms. We were able to confirm that the first band is indeed the dimer of Mn-SOD, as it is not detected in extracts from *sod-2(gk257)* null mutants and is susceptible to disruption by strong denaturing conditions such as 6 M guanidine hydrochloride (Supplementary Fig. S6). (C) *sod-2* OE results in an ~ 20% increase in monomeric Mn-SOD, but almost a threefold increase in dimeric Mn-SOD. **P < 0.01 vs control (*rol-6*) in each case; mean of three trials. Actin was used as a protein loading control. (D) Mn-SOD activity in *sod-2* OE lines. SOD overexpression leads to a 50% increase in SOD-2 activity relative to controls (*rol-6*). *P < 0.05 vs control (*rol-6*).

**Fig. 4:**
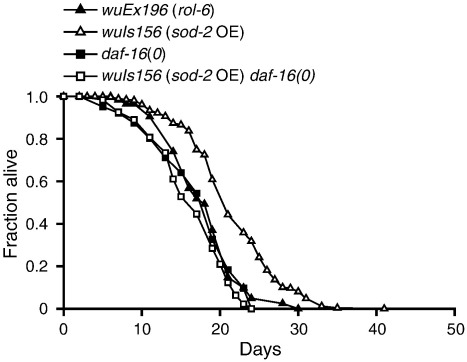
Life-span extension by Mn-SOD is *daf-16* dependent. Life-span curve showing that *sod-2* OE does not extend life span in a *daf-16(mgDf50)* mutant background. A representative trial is depicted; for full life-span data, including statistics, means, and median values, see Supplementary Table S2.

*sod-2* OE extended mean and maximum life span by approximately 25% (Fig. 3A; Supplementary Table S2). The magnitude of this effect is comparable to that seen from *sod-1* OE in trials performed at the same time (Supplementary Table S1). We verified that elevated *sod-2* gene copy number increased SOD-2 protein levels using Western blots (Figs. 3B and C and Supplementary Fig. S6). We also observed a 1.5-fold increase in Mn-SOD activity in the *sod-2* OE strain (Fig. 3D). Like *sod-1,* overexpression of *sod-2* did not reduce levels of protein oxidation, but it did not increase them, either (Supplementary Fig. S7). As for *sod-1* OE, the life-span increase by *sod-2* OE was *daf-16*-dependent (Fig. 4; Supplementary Table S2).

### sod-1 OE elevates H_2_O_2_ in vivo but longevity is not reduced by catalase co-overexpression

**Fig. 5:**
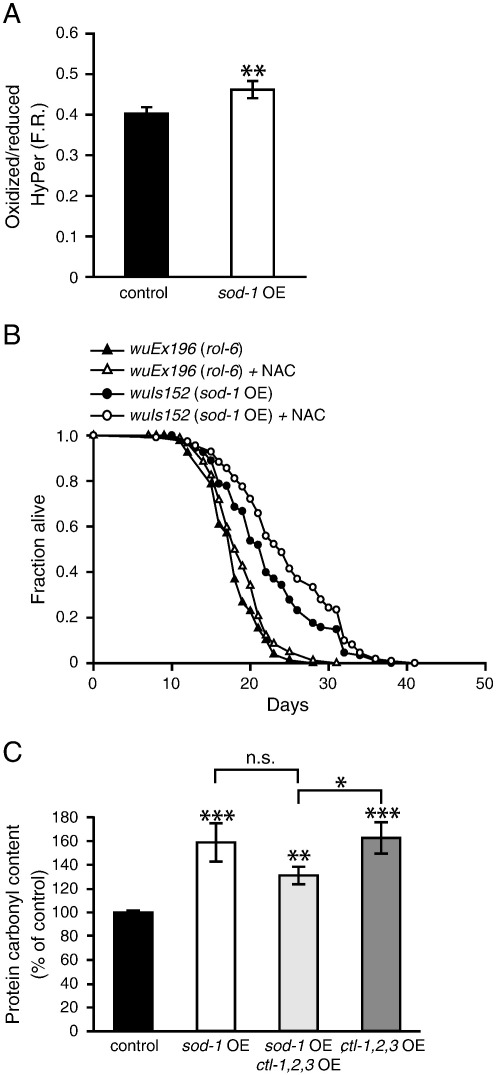
H_2_O_2_ increase by *sod-1* OE might not be the mediator of the effects on life span. (A) In vivo H_2_O_2_ measurements using HyPer in *sod-1* OE line bearing *wuIs152. sod-1* OE increases steady-state levels of H_2_O_2_. *P < 0.05 vs HyPer control. (B) 1 mM NAC treatment does not suppress extended life span of *sod-1* OE worms. 5 mM NAC greatly reduced life span in both *sod-1* OE and control worms (data not shown). A representative trial is depicted; for full life-span data, including statistics, means, and median values, see Supplementary Table S2. (C) Catalase co-overexpression (co-OE) does not significantly reduce protein oxidation resulting from *sod-1* OE. n.s., *P* > 0.05 for *sod-1* OE vs *sod-1;* catalase co-OE (*sod-1* OE; *ctl*-*1,2,3* OE) (*n* = 3). The co-OE line shows increased protein oxidation relative to the control strain, **P < 0.01 vs control (*rol-6*) *P < 0.05 and ***P < 0.001.

Because SOD activity generates H_2_O_2_, we explored the possibility that elevated levels of H_2_O_2_ in SOD OE lines might be the cause of the increase in life span. We first measured steady-state levels of H_2_O_2_ production in vivo using a genetically encoded H_2_O_2_-specific fluorescent probe (see Materials and methods). This revealed a modest but statistically significant increase in steady-state H_2_O_2_ levels in *sod-1* OE animals (Fig. 5A). Amplex red assays of worm lysates confirmed increased H_2_O_2_ levels in *sod-1* OE relative to controls (Supplementary Fig. S8A).

However, we previously noted that the slight life-span increase of *sod-1* overexpressing animals appeared unaffected by catalase co-overexpression (co-OE) [11], which argues against a role for H_2_O_2_ in inducing the DAF-16-dependent life-span extension. We reinvestigated this question and again found that catalase co-OE did not suppress longevity in two *sod-1* OE strains (Supplementary Fig. S8B and Table S2). Furthermore, the increase in protein carbonylation in SOD-1 OE animals was not reduced to wild-type levels by catalase co-OE (Fig. 5C). We confirmed that catalase OE lowers H_2_O_2_ levels (Supplementary Fig. S8A).

**Fig. 6:**
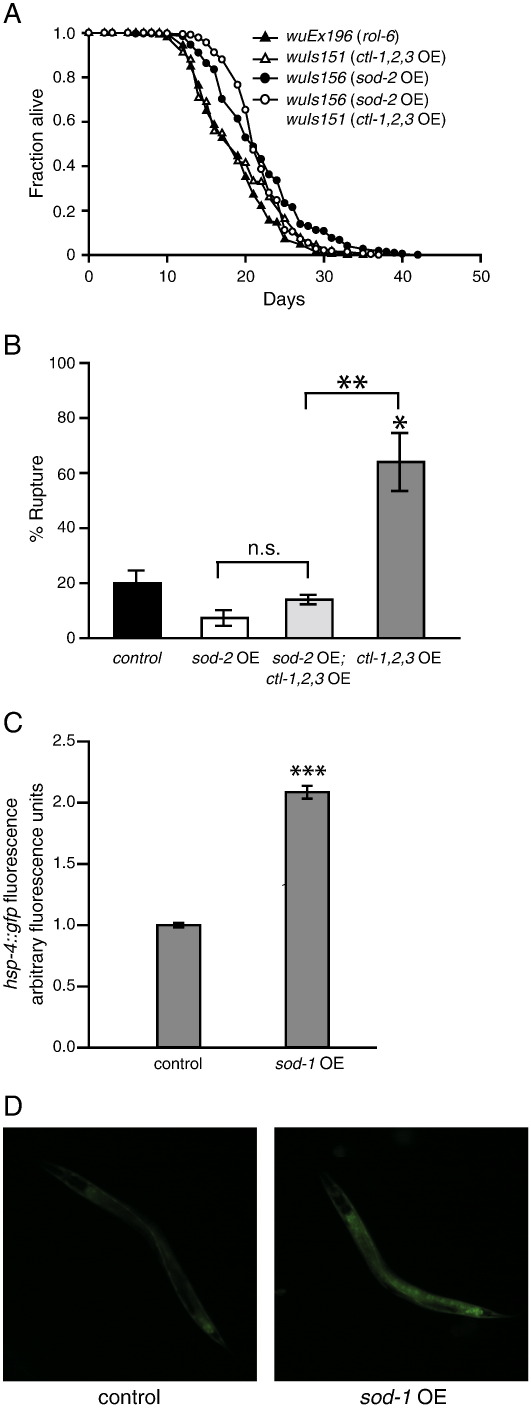
H_2_O_2_ might not be the cause of life-span extension induced by *sod-2* OE. (A) Catalase co-OE does not suppress the life-span extension of *sod-2* OE animals. A representative trial is depicted; for full life-span data, including statistics, means, and median values, see Supplementary Table S2. (B) Rupture phenotype caused by catalase OE is suppressed by *sod-2* OE. (C) Overexpression of *sod-1* induces expression of *hsp-4::gfp,* a marker for ER stress. (D) Epifluorescence images of L4 animals carrying *hsp-4::gfp* in both wild-type and *sod-1* overexpressing lines. *P < 0.05, **P < 0.01, ***P < 0.001.

One possibility is that differences in the localization of the overexpressed SOD and catalase might account for the observed lack of suppression of *sod-1* OE longevity. To probe this, we used NAC (1 mM), which can act as a broad-spectrum antioxidant, but again *sod-1* OE longevity was not suppressed (Fig. 5B, Supplementary Table S2). Catalase co-OE did not suppress *sod-2* OE longevity, either (Fig. 6A, Supplementary Table S2). Catalase OE alone caused high levels of mortality due to rupture (extrusion of the uterus through the vulva) (Fig. 6B), potentially reflecting deleterious effects of H_2_O_2_ deficiency. Co-OE of *sod-2* fully suppressed this mortality (Fig. 6B), consistent with restoration of H_2_O_2_ levels. We conclude that *sod-1* OE elevates steady-state H_2_O_2_ levels in vivo, but we could find no evidence that this causes the increase in life span.

### sod-1 OE elevates expression of markers of the unfolded protein response

Cu/Zn-SOD is a highly expressed cytosolic protein [11] and Cu/Zn-SOD protein levels increase approximately sevenfold in *sod-1* OE lines (Supplementary Fig. S4). Potentially, this large increase in protein levels could upset protein folding homeostasis, e.g., by increasing levels of unfolded proteins in the endoplasmic reticulum (ER). Such ER stress can induce an unfolded protein response (UPR) to restore protein folding homeostasis. Expression of *hsp-4,* encoding an ER chaperone protein, is induced under conditions of ER stress [38], [39]. *sod-1* OE increased expression of *hsp-4::gfp* (Figs. 6C and D), and also of *hsp-6::gfp,* a marker of the mitochondrial UPR (Supplementary Fig. S9A). Induction of the UPR can also increase thermotolerance, but we detected no difference between *sod-1* OE and wild-type worms in resistance to heat stress (35 °C) (data not shown).

In *C. elegans,* IRE-1, PEK-1, and ATF-6 can sense ER stress and activate a UPR [40], [41]. IRE-1 activation leads to increased levels of the XBP-1 transcription factor, which promotes expression of genes that protect against ER stress [39], [42]. RNAi of *ire-1* and *xbp-1* markedly reduced *hsp-4::gfp* expression in both the presence and the absence of *sod-1* OE (Supplementary Fig. S9B), whereas that of *pek-1* and *atf-6* did not (data not shown). *ire-1* and *xbp-1* RNAi did not fully abrogate the induction of *hsp-4::gfp* expression by *sod-1* OE, but reduced its magnitude in terms of fold change increase (Supplementary Fig. S9C). This could reflect either incomplete knockdown of *ire-1* and *xbp-1* by RNAi or a non-*ire-1/xbp-1-*dependent component of the effects of *sod-1* OE on *hsp-4::gfp* expression. Overall, these results suggest that *sod-1* OE induces an ER stress response. RNAi of *xbp-1* and *ire-1* also reduced the life span of *sod-1* OE animals more than that of wild-type animals in two independent trials (Supplementary Fig. S10 and Table S2). This result is consistent with a role for the UPR in mediating the life-span extension by *sod-1* OE.

## Discussion

### Effect of sod-1 OE on life span is not due to enhanced antioxidant capacity

We previously reported that overexpression of the *sod-1* cytosolic Cu/Zn-SOD increases *C. elegans* life span and proposed that this was due to reduced levels of cytosolic O_2_^•−^[11]. However, this now seems unlikely, because *sod-1* OE reduces resistance to oxidative stress [11], increases protein oxidation and steady-state levels of H_2_O_2_, and extends life span only in the presence of the DAF-16/FoxO transcription factor*.* This is consistent with earlier observations that enhancing SOD activity in vivo by administration of SOD mimetics does not extend *C. elegans* life span [3], [4], [5], [43]. These results are also consistent with the finding that Mn-SOD overexpression in *Drosophila* causes changes in gene expression that are similar to those found in *C. elegans* insulin/IGF-1 signaling mutants [44], which also require DAF-16. Although the FoxO dependence of the *Drosophila* life-span extension has not been tested, it seems possible that life-span extension involves mechanisms similar to those in *C. elegans.*

Our results illustrate how overexpressing antioxidant genes can produce complex effects and have unexpected consequences, and emphasize the importance of interpreting such studies with care [45].

### How does sod-1 OE increase life span?

We explored alternative mechanisms by which *sod-1* OE might increase life span. First, we showed that *sod-1* OE increases steady-state levels of H_2_O_2_ in vivo. However, results of catalase co-overexpression tests did not support the view that elevated H_2_O_2_ levels increase life span.

Expression studies imply that SOD-1 is an abundant cytosolic protein [11]. Thus, overexpression of *sod-1* might lead to levels of SOD-1 protein so high as to overwhelm the cellular protein folding machinery, inducing a UPR. Consistent with this, *sod-1* OE increased expression of *hsp-4::gfp* and *hsp-6::gfp,* markers of the cytosolic and mitochondrial UPR, respectively. The increase in *hsp-4::gfp* expression was partially suppressed by RNAi of *ire-1* or *xbp-1,* which also partially suppressed the longevity of *sod-1* OE worms. This is consistent with the involvement of an ER stress response in the longevity of *sod-1* OE worms. *ire-1* and *xbp-1* have previously been shown to promote longevity: mutation of either gene reduces life span in a *daf-2(e1370)* mutant [38]. Thus, *sod-1* OE might increase life span by triggering a UPR.

### The role of daf-16 in sod-1 OE-induced longevity

The longevity induced by overexpression of *sod-1* and *sod-2* requires the presence of *daf-16,* which is also required for the longevity of mutants with reduced IIS [31]. This could imply that similar longevity mechanisms are operative in *sod* OE strains and long-lived IIS mutants, i.e., that similar *daf-16*-dependent changes in gene expression are operative. However, certain observations suggest that this might not be so. *sod-3* is a direct transcriptional target of DAF-16 that shows *daf-16*-dependent up-regulation in *daf-2* mutants [46], [47], [48]. However, neither *sod-3* or *mtl-1* (another *daf-16* target) [49] is up-regulated in *sod-1* OE lines [50]. Moreover, and surprisingly, the increase in protein oxidation caused by *sod-1* OE also proved to be *daf-16* dependent (Supplementary Fig. S5A). We also confirmed that protein oxidation levels are not increased in *daf-2* mutants or, in *daf-2* mutants, reduced by mutation of *daf-16* (Supplementary Fig. S5B), consistent with a previous study [51]. These observations suggest that in the different contexts of *sod-1* OE and mutation of *daf-2,* DAF-16 might act in a different way to increase life span.

The *daf-16-*dependent increase in protein levels in *sod-1* OE worms also implies that, in this context at least, DAF-16 does not increase life span by reducing levels of protein oxidation. Moreover, it provides further evidence that increasing levels of protein oxidation does not necessarily accelerate aging. Likewise, deletion of the *sod-2* Mn-SOD increases oxidative damage levels but does not reduce life span [13].

## Conclusions

The results presented here imply that the increased life span caused by *sod-1* and *sod-2* overexpression is not a consequence of lowered levels of ROS or of oxidative damage. This means that the increased life span of these strains may not be taken as evidence in support of the oxidative damage theory of aging. In fact, the association of increased levels of protein oxidation with increased life span argues against the theory. The exact mechanism by which *sod* overexpression triggers a *daf-16*-dependent increase in life span remains unclear. However, our results suggest that high levels of SOD protein may trigger an unfolded protein response that contributes to the increase in life span.

## References

[1] Beckman K.B., Ames B.N. (1998). The free radical theory of aging matures. Physiol. Rev..

[2] Harman D. (1956). Aging—a theory based on free-radical and radiation-chemistry. J. Gerontol..

[3] Van Raamsdonk J.M., Hekimi S. (2010). Reactive oxygen species and aging in Caenorhabditis elegans: causal or casual relationship?. Antioxid. Redox Signal..

[4] Gems D., Doonan R. (2009). Antioxidant defense and aging in C. elegans: is the oxidative damage theory of aging wrong?. Cell Cycle.

[5] Keaney M., Matthijssens F., Sharpe M., Vanfleteren J., Gems D. (2004). Superoxide dismutase mimetics elevate superoxide dismutase activity in vivo but do not retard aging in the nematode Caenorhabditis elegans. Free Radic. Biol. Med..

[6] Kim J., Takahashi M., Shimizu T., Shirasawa T., Kajita M., Kanayama A., Miyamoto Y. (2008). Effects of a potent antioxidant, platinum nanoparticle, on the lifespan of Caenorhabditis elegans. Mech. Ageing Dev..

[7] Uchiyama S., Koike H., Shimizu T., Shirasawa T. (2005). A superoxide dismutase/catalase mimetic extends the lifespan of short-lived mev-1 mutant but not the wild type strain in Caenorhabditis elegans. Anti Aging Med. Res..

[8] Heidler T., Hartwig K., Daniel H., Wenzel U. (2010). Caenorhabditis elegans lifespan extension caused by treatment with an orally active ROS-generator is dependent on DAF-16 and SIR-2.1. Biogerontology.

[9] Yang W., Hekimi S. (2010). A mitochondrial superoxide signal triggers increased longevity in Caenorhabditis elegans. PLoS Biol..

[10] Lee S.J., Hwang A.B., Kenyon C. (2010). Inhibition of respiration extends C. elegans life span via reactive oxygen species that increase HIF-1 activity. Curr. Biol..

[11] Doonan R., McElwee J.J., Matthijssens F., Walker G.A., Houthoofd K., Back P., Matscheski A., Vanfleteren J.R., Gems D. (2008). Against the oxidative damage theory of aging: superoxide dismutases protect against oxidative stress but have little or no effect on life span in Caenorhabditis elegans. Genes Dev..

[12] Honda Y., Tanaka M., Honda S. (2008). Modulation of longevity and diapause by redox regulation mechanisms under the insulin-like signaling control in Caenorhabditis elegans. Exp. Gerontol..

[13] Van Raamsdonk J.M., Hekimi S. (2009). Deletion of the mitochondrial superoxide dismutase sod-2 extends lifespan in Caenorhabditis elegans. PLoS Genet..

[14] Yang W., Li J., Hekimi S. (2007). A Measurable increase in oxidative damage due to reduction in superoxide detoxification fails to shorten the life span of long-lived mitochondrial mutants of Caenorhabditis elegans. Genetics.

[15] Yen K., Patel H.B., Lublin A.L., Mobbs C.V. (2009). SOD isoforms play no role in lifespan in ad lib or dietary restricted conditions, but mutational inactivation of SOD-1 reduces life extension by cold. Mech. Ageing Dev..

[16] Perez V.I., Bokov A., Van Remmen H., Mele J., Ran Q., Ikeno Y., Richardson A. (2009). Is the oxidative stress theory of aging dead?. Biochim. Biophys. Acta.

[17] Sun J., Tower J. (1999). FLP recombinase-mediated induction of Cu/Zn-superoxide dismutase transgene expression can extend the life span of adult Drosophila melanogaster flies. Mol. Cell. Biol..

[18] Ford D., Hoe N., Landis G.N., Tozer K., Luu A., Bhole D., Badrinath A., Tower J. (2007). Alteration of Drosophila life span using conditional, tissue-specific expression of transgenes triggered by doxycycline or RU486/Mifepristone. Exp. Gerontol..

[19] Huang T.T., Carlson E.J., Gillespie A.M., Shi Y., Epstein C.J. (2000). Ubiquitous overexpression of CuZn superoxide dismutase does not extend life span in mice. J. Gerontol. A Biol. Sci. Med. Sci..

[20] Karanjawala Z.E., Murphy N., Hinton D.R., Hsieh C.L., Lieber M.R. (2002). Oxygen metabolism causes chromosome breaks and is associated with the neuronal apoptosis observed in DNA double-strand break repair mutants. Curr. Biol..

[21] Rando T.A., Crowley R.S., Carlson E.J., Epstein C.J., Mohapatra P.K. (1998). Overexpression of copper/zinc superoxide dismutase: a novel cause of murine muscular dystrophy. Ann. Neurol..

[22] Buettner G.R., Ng C.F., Wang M., Rodgers V.G.J., Schafer F.Q. (2006). A new paradigm: manganese superoxide dismutase influences the production of H_2_O_2_ in cells and thereby their biological state. Free Radic. Biol. Med..

[23] Olahova M., Taylor S.R., Khazaipoul S., Wang J., Morgan B.A., Matsumoto K., Blackwell T.K., Veal E.A. (2008). A redox-sensitive peroxiredoxin that is important for longevity has tissue- and stress-specific roles in stress resistance. Proc. Natl. Acad. Sci. U. S. A..

[24] Loh K., Deng H., Fukushima A., Cai X., Boivin B., Galic S., Bruce C., Shields B.J., Skiba B., Ooms L.M., Stepto N., Wu B., Mitchell C.A., Tonks N.K., Watt M.J., Febbraio M.A., Crack P.J., Andrikopoulos S., Tiganis T. (2009). Reactive oxygen species enhance insulin sensitivity. Cell Metab..

[25] Ristow M., Zarse K., Oberbach A., Kloting N., Birringer M., Kiehntopf M., Stumvoll M., Kahn C.R., Bluher M. (2009). Antioxidants prevent health-promoting effects of physical exercise in humans. Proc. Natl. Acad. Sci. U. S. A..

[26] Gems D., Partridge L. (2008). Stress-response hormesis and aging: "that which does not kill us makes us stronger.". Cell Metab..

[27] Cypser J.R., Johnson T.E. (2002). Multiple stressors in Caenorhabditis elegans induce stress hormesis and extended longevity. J. Gerontol. A Biol. Sci. Med. Sci..

[28] Hartwig K., Heidler T., Moch J., Daniel H., Wenzel U. (2009). Feeding a ROS-generator to Caenorhabditis elegans leads to increased expression of small heat shock protein HSP-16.2 and hormesis. Genes Nutr..

[29] Lithgow G.J., White T.M., Melov S., Johnson T.E. (1995). Thermotolerance and extended life-span conferred by single-gene mutations and induced by thermal stress. Proc. Natl. Acad. Sci. U. S. A..

[30] Cypser J.R., Johnson T.E. (2003). Hormesis in Caenorhabditis elegans dauer-defective mutants. Biogerontology.

[31] Kenyon C., Chang J., Gensch E., Rudner A., Tabtiang R. (1993). A C. elegans mutant that lives twice as long as wild-type. Nature.

[32] Sulston J., Hodgkin J. (1988). Methods: in the Nematode Caenorhabditis elegans.

[33] Gems D., Sutton A.J., Sundermeyer M.L., Albert P.S., King K.V., Edgley M.L., Larsen P.L., Riddle D.L. (1998). Two pleiotropic classes of daf-2 mutation affect larval arrest, adult behavior, reproduction and longevity in Caenorhabditis elegans. Genetics.

[34] Belousov V.V., Fradkov A.F., Lukyanov K.A., Staroverov D.B., Shakhbazov K.S., Terskikh A.V., Lukyanov S. (2006). Genetically encoded fluorescent indicator for intracellular hydrogen peroxide. Nat. Methods.

[35] Comporti M. (1998). Lipid peroxidation and biogenic aldehydes: from the identification of 4-hydroxynonenal to further achievements in biopathology. Free. Radic. Res..

[36] Monnier V.M., Sell D.R., Nagaraj R.H., Miyata S., Grandhee S., Odetti P., Ibrahim S.A. (1992). Maillard reaction-mediated molecular damage to extracellular matrix and other tissue proteins in diabetes, aging, and uremia. Diabetes.

[37] Grune T., Reinheckel T., Davies K.J. (1997). Degradation of oxidized proteins in mammalian cells. FASEB J..

[38] Henis-Korenblit S., Zhang P.C., Hansen M., McCormick M., Lee S.J., Cary M., Kenyon C. (2010). Insulin/IGF-1 signaling mutants reprogram ER stress response regulators to promote longevity. Proc. Natl. Acad. Sci. U. S. A..

[39] Calfon M., Zeng H.Q., Urano F., Till J.H., Hubbard S.R., Harding H.P., Clark S.G., Ron D. (2002). IRE1 couples endoplasmic reticulum load to secretory capacity by processing the XBP-1 mRNA. Nature.

[40] Mori K. (2009). Signalling pathways in the unfolded protein response: development from yeast to mammals. J. Biochem..

[41] Shen X., Ellis R.E., Sakaki K., Kaufman R.J. (2005). Genetic interactions due to constitutive and inducible gene regulation mediated by the unfolded protein response in C. elegans. PLoS Genet..

[42] Shen X., Ellis R.E., Lee K., Liu C.Y., Yang K., Solomon A., Yoshida H., Morimoto R., Kurnit D.M., Mori K., Kaufman R.J. (2001). Complementary signaling pathways regulate the unfolded protein response and are required for C. elegans development. Cell.

[43] Keaney M., Gems D. (2003). No increase in lifespan in *Caenorhabditis elegans* upon treatment with the superoxide dismutase mimetic EUK-8. Free Radic. Biol. Med..

[44] Curtis C., Landis G.N., Folk D., Wehr N.B., Hoe N., Waskar M., Abdueva D., Skvortsov D., Ford D., Luu A., Badrinath A., Levine R.L., Bradley T.J., Tavare S., Tower J. (2007). Transcriptional profiling of MnSOD-mediated lifespan extension in Drosophila reveals a species-general network of aging and metabolic genes. Genome Biol..

[45] Murphy M.P., Holmgren A., Larsson N.G., Halliwell B., Chang C.J., Kalyanaraman B., Rhee S.G., Thornalley P.J., Partridge L., Gems D., Nystrom T., Belousov V., Schumacker P.T., Winterbourn C.C. (2011). Unraveling the biological roles of reactive oxygen species. Cell Metab..

[46] Honda Y., Honda S. (1999). The daf-2 gene network for longevity regulates oxidative stress resistance and Mn-superoxide dismutase gene expression in Caenorhabditis elegans. FASEB J..

[47] Libina N., Berman J.R., Kenyon C. (2003). Tissue-specific activities of *C. elegans* DAF-16 in the regulation of lifespan. Cell.

[48] Oh S.W., Mukhopadhyay A., Dixit B.L., Raha T., Green M.R., Tissenbaum H.A. (2006). Identification of direct DAF-16 targets controlling longevity, metabolism and diapause by chromatin immunoprecipitation. Nat. Genet..

[49] Barsyte D., Lovejoy D.A., Lithgow G.J. (2001). Longevity and heavy metal resistance in daf-2 and age-1 long-lived mutants of Caenorhabditis elegans. FASEB J..

[50] Back P., Matthijssens F., Vlaeminck C., Braeckman B.P., Vanfleteren J.R. (2010). Effects of sod gene overexpression and deletion mutation on the expression profiles of reporter genes of major detoxification pathways in Caenorhabditis elegans. Exp. Gerontol..

[51] Yasuda K., Adachi H., Fujiwara Y., Ishii N. (1999). Protein carbonyl accumulation in aging dauer formation-defective (daf) mutants of Caenorhabditis elegans. J. Gerontol. A Biol. Sci. Med. Sci..

[52] Trinh C.H., Hunter T., Stewart E.E., Phillips S.E., Hunter G.J. (2008). Purification, crystallization and X-ray structures of the two manganese superoxide dismutases from Caenorhabditis elegans. Acta Crystallogr. Sect. F Struct. Biol. Cryst. Commun..

