# Supplementary material for: Increased life span from overexpression of superoxide dismutase in Caenorhabditis elegans is not caused by decreased oxidative damage
Source: Free Radic Biol Med. 2011 Oct 15;51(8):1575–82. doi: 10.1016/j.freeradbiomed.2011.07.020 (PMC3202636; doi:10.1016/j.freeradbiomed.2011.07.020)
Supplement: Supplementary file 1 — Supplementary materials [file mmc1.pdf]

Material and Methods supplemental

Assay of 4-HNE-protein adducts

The level of 4-HNE-modified proteins was determined by ELISA using the OxiSelect™ HNE-His Adduct ELISA Kit according to the manufacturer's instructions (Cell biolabs). 50 µg/ml of total protein extracts were adsorbed onto a 96-well plate at 4°C overnight. The absorbance of each well was read using a microplate reader (Infinite 200, TECAN) using 450 nm as the primary wavelength.

Determination of *in vitro* proteasome activities

Peptidase activities of the proteasome were assayed using the fluorogenic peptide succinyl-Leu-Leu-Val-Tyr-amidomethylcoumarin (LLVY-AMC) (Biomol) for the chymotrypsin-like (CT-L) activity, Boc-Leu-Arg-Arg-AMC (LRR-AMC) for the trypsin-like activity (T-L) and Z-Leu-Leu-Glu-AMC (LLE-AMC) for the caspase-like activity. The mixture, containing 50 µg of crude protein cellular extracts in proteasome buffer (25 mM Tris-HCl, pH 7.5), was incubated at 37°C with either the substrate LLVY-AMC at 25 µM, LLE-AMC at 300µM or LRR-AMC at 80µM in a final volume of 200 µl. Enzymatic kinetics were conducted in a temperature-controlled microplate reader (Infinite 200, TECAN). Excitation/emission wavelengths were 350/440 nm for aminomethylcoumarin. Proteasome activities were determined as the difference between total activity and the remaining activity of the crude extract in the presence of 20 µM proteasome inhibitor MG132.

Measurement of hydrogen peroxide in worm lysates

H_2O_2 was measured in worm protein extracts. Briefly, 200 worms were individually picked, washed to remove contaminant bacteria and homogenized in CelLytic buffer using a Bioruptor. Oxidation of 50 µM Amplex Red to highly fluorescent product resorufin in the presence of 0.1 U/mL HRP was measured after 30 min of incubation using a microplate reader (Infinite 200, TECAN). Excitation and emission wavelengths of 530 and 590 nm, respectively, were used. The

oxidation of Amplex Red, in the absence of protein extract, was negligible. Obtained values were normalized by the total protein content of each sample.

Supplemental figure legends

Fig. S1 SOD-1 over-expression (OE) does not reduce lipid peroxidation or glycation products. (A) 4-HNE- protein adducts are not decreased by *sod-1* OE. Adducts were determined by ELISA. (B) Advanced glycation end products are not reduced by *sod-1* OE. Left panel: Immunoblot showing carboxymethyllysine (CML)-modified proteins. Right panel: Quantified changes in total CML content. While no statistically significant differences were found between the two strains, the data suggests that levels of these forms of molecular damage might be modestly increased by *sod-1* OE. Additional trials would be necessary to establish whether this is the case. The data in bar graphs represent means (n = 3) ± S.E.

Fig. S2 *sod-1* OE does not alter proteasome activities. The proteasome chymotrypsin-like (CT-L), caspase-like (C-L) and trypsin-like (T-L) activity were assayed *in vitro* using the fluorogenic peptide substrates LLVY-AMC, LLE-AMC and LSTR-AMC, respectively (n=4). No statistically significant differences were found between control (*rol-6*) and *sod-1* OE strains. However, *daf-16(0)* strains contained less proteasome 20S catalytic activity. *P < 0.05 vs. control (*rol-6*) (Student's paired t test). This could imply that DAF-16 acts as an activator of proteasome activity.

Fig. S3 *sod-1* OE longevity is not AMPK dependent. *sod-1* OE robustly increases lifespan in an *aak-2(ok524)* background. The figure depicts a representative trial; for full lifespan data, including statistics, mean and median values, see Table S1. Inlay: Representative western blot (of 3 independent trials) for phosphorylated-AMPK at Thr-172 (P-AAK-2), and actin loading control. *sod-1* OE did not detectably increase AAK-2 phosphorylation.

Fig. S4 *daf-16(0)* does not suppress over-expression of *sod-1*. (B) Quantification of the total SOD-1 content in each strain (n = 3). SOD-1 protein levels are increased ~7-fold, independently of *daf-16,* relative to the control, ***P < 0.001 vs. control (*rol-6*) (Student's t test). No statistically significant changes were found when comparing the two *sod-1* OE strains. Data in bar graphs represent means ± S.E.

Fig. S5 The increase in protein oxidation resulting from *sod-1* over-expression is *daf-16* dependent. (A) *daf-16(mgDf50)* fully suppresses the increase in protein carbonyl levels resulting from *sod-1* OE. (B) Protein carbonyl levels are not increased by *daf-2(e1370)*, or reduced in *daf-16; daf-2* double mutants. This result is consistent with a previous study, which found no difference in protein carbonyl levels between wild type (N2) and *daf-2* mutants before day 20 of adulthood [48].

Fig. S6 Guanidine hydrochloride disrupts MnSOD dimer and increases ratio of MnSOD monomers to dimers. (A) Immunoblot showing the relative protein levels of SOD-2 (MnSOD) under strong denaturing conditions. (B) Quantification of relative levels of monomeric and dimeric SOD-2.

Fig. S7 No changes in protein oxidation by *sod-2* OE. Left panel: representative Oxyblot showing changes in oxidized protein content. Right panel: quantification of the carbonyl content (n = 3). Data in bar graphs represent means ± S.E.

Fig. S8 Evidence that H_2O_2 increase by *sod-1* overexpression does not mediate the effect in lifespan. (A) H_2O_2 production in worm lysates. Results from Amplex red/horseradish peroxidase assays show a significantly increase in H_2O_2 production in *sod-1* OE worm extracts, as shown by the significant increases in resorufin fluorescence (n=3). A significant decrease in H_2O_2 production is observed in *ctl-1,2,3* OE worms. *P < 0.05, **P < 0.01 vs. control (*rol-6*). (B) *sod-1* OE longevity is not suppressed by co-over-expression of catalase. See Table S2 for lifespan statistics, mean and median values.

Fig. S9 *sod-1* OE increases expression of *hsp-4::gfp* and *hsp-6::gfp* (A) *sod-1* OE increases expression of *hsp-6::gfp*. One additional control line and two additional *sod-1* OE lines were also

tested with similar results. (B) RNAi of *ire-1* and *xbp-1* reduces *hsp-4::gfp* expression both without or with over-expression of *sod-1*. (C) RNAi of *ire-1* and *xbp-1* reduces the magnitude of the fold-change increase in *hsp-4::gfp* expression induced by *sod-1* OE. afu (arbitrary fluorescence units)

Fig. S10 Loss of *ire-1* or *xbp-1* reduces the lifespan of *sod-1* OE more than wild type animals. A, B, trial [8], B, C trial [9], see Table S2. Non-RNAi controls were maintained on E. coli containing the L4440 empty vector.

FigS1

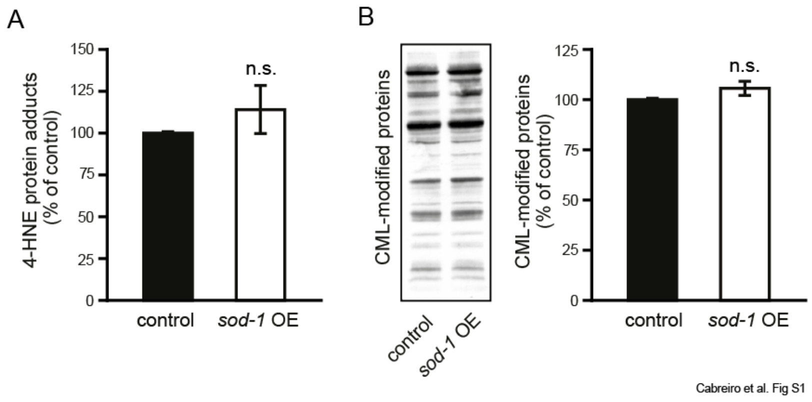
A
4-HNE protein adducts (% of control)
150
125
100
75
50
25
0
n.s.
control
sod-1 OE
B
CML-modified proteins
control
sod-1 OE
CML-modified proteins (% of control)
125
100
75
50
25
0
n.s.
control
sod-1 OE
Cabreiro et al. Fig S1

Fig S2

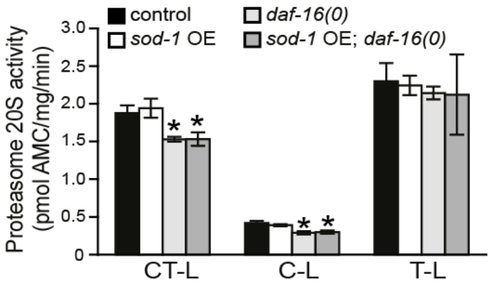

Cabreiro et al. Fig S2

Fig S3

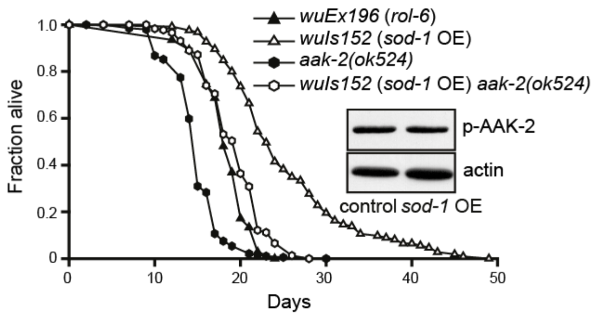

Cabreiro et al. Fig S3

Fig S4

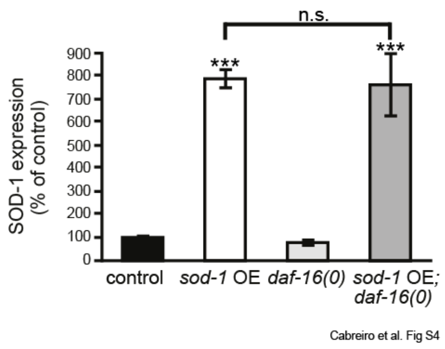
n.s.
SOD-1 expression
(% of control)
900
800
700
600
500
400
300
200
100
0
***
***
control
sod-1 OE
daf-16(0)
sod-1 OE;
daf-16(0)
Cabreiro et al. Fig S4

Fig S5

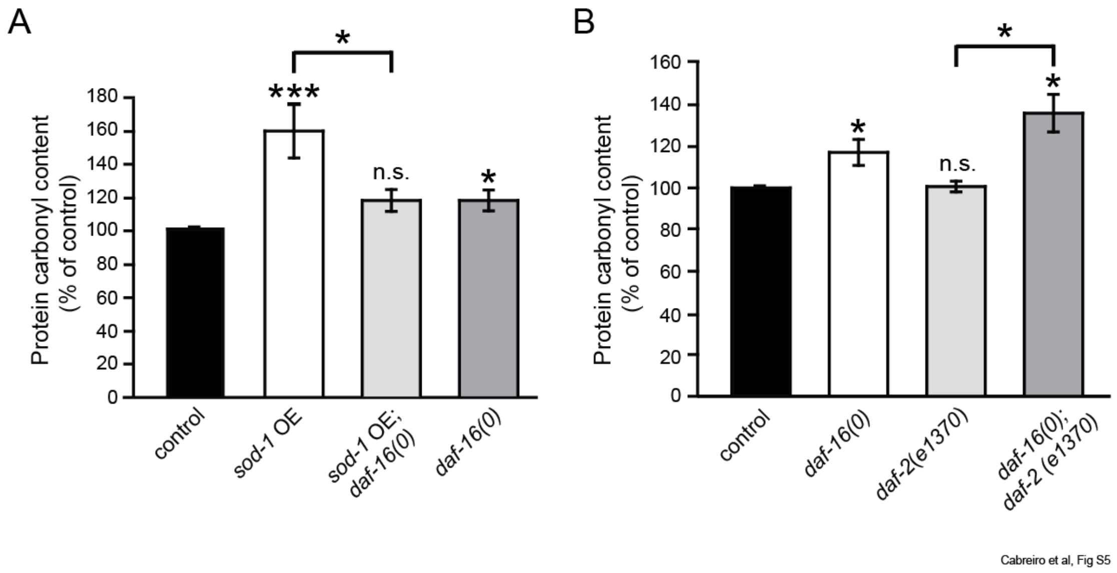
A
*
***
n.s.
*
180
160
140
120
100
80
60
40
20
0
Protein carbonyl content (% of control)
control
sod-1 OE
sod-1 OE; daf-16(0)
daf-16(0)
B
*
*
*
n.s.
160
140
120
100
80
60
40
20
0
Protein carbonyl content (% of control)
control
daf-16(0)
daf-2(e1370)
daf-16(0); daf-2 (e1370)
Cabreiro et al, Fig S5

Fig S6

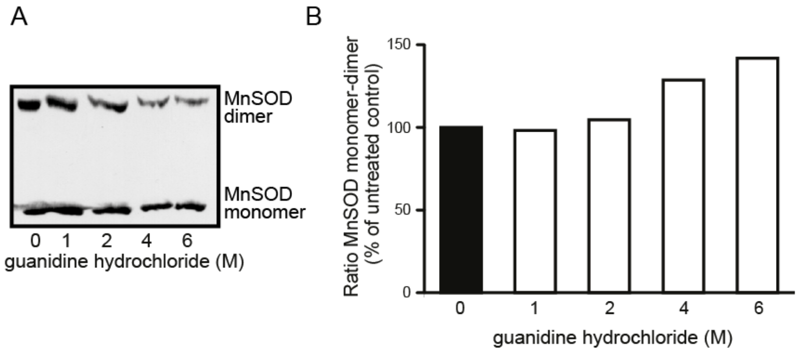

Cabreiro et al. Fig S6

Fig S7

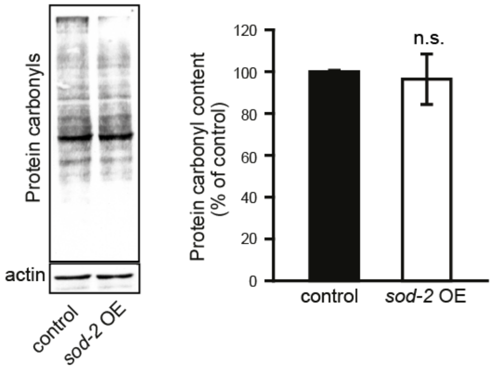

Cabreiro et al. Fig S7

Fig S8

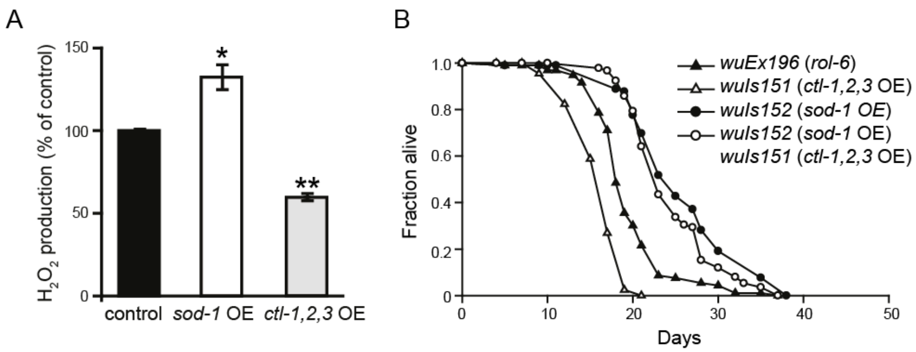

Cabreiro et al. Fig S8

Fig S9

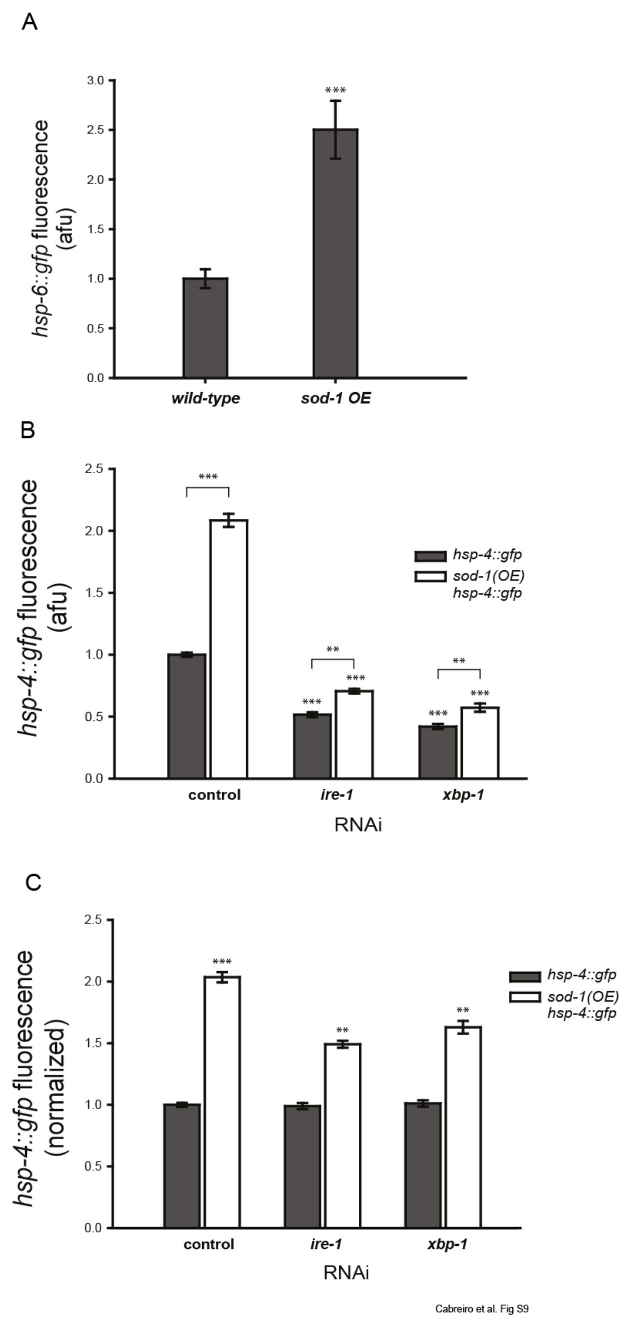
A
hsp-6::gfp fluorescence (afu)
3.0
2.5
2.0
1.5
1.0
0.5
0.0
***
wild-type
sod-1 OE
B
hsp-4::gfp fluorescence (afu)
2.5
2.0
1.5
1.0
0.5
0.0
hsp-4::gfp
sod-1(OE) hsp-4::gfp
***
**
**
control
ire-1
xbp-1
RNAi
C
hsp-4::gfp fluorescence (normalized)
2.5
2.0
1.5
1.0
0.5
0.0
hsp-4::gfp
sod-1(OE) hsp-4::gfp
***
**
**
control
ire-1
xbp-1
RNAi
Cabreiro et al. Fig S9

Fig S10

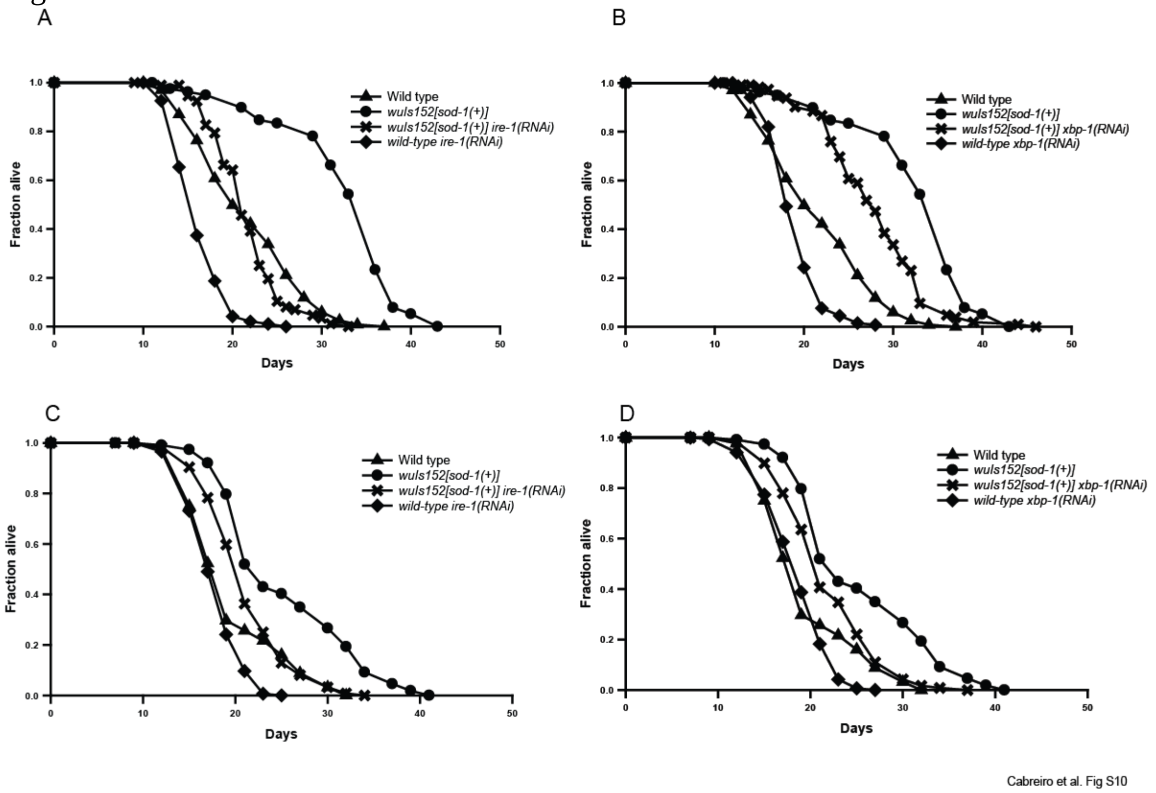
A
B
C
D
Fraction alive
Days
Wild type
wuIs152[sod-1(+)]
wuIs152[sod-1(+)] ire-1(RNAi)
wild-type ire-1(RNAi)
wuIs152[sod-1(+)] xbp-1(RNAi)
wild-type xbp-1(RNAi)
Cabreiro et al. Fig S10

Table S1: Survival data

Genotype [Trial number]	n	Mean Lifespan	Median Lifespan	% Change[a]	P-value[b]
SEPARATE TRIALS					
rol-6(dm) [1]	117	18.3	18		
wuIs152 (sod-1 OE*)* [1]	119	25.2	23	+28	<0.0001
wuEx123 (sod-1 OE*)* [1]	120	23.0	22	+22	<0.0001
rol-6(dm) [2]	72	18.9	18		
wuIs152 (sod-1 OE*)* [2]	50	*n.d.*	26	+44	<0.0001
daf-16(mgDf50) [1]	116	15.2	16		
wuEx123 (sod-1 OE*) daf-16(mgDf50)* [1]	119	15.9	16		0.48
wuIs152 (sod-1 OE*) daf-16(mgDf50)* [1]	92	13.9	14	-13	<0.0001
wuIs152 (sod-1 OE*)* [3]	74	29.3	30		
daf-16(mgDf50) [3]	121	17.6	18		
wuIs152 (sod-1 OE*) daf-16(mgDf50)* [3]	108	15.9	16	-11	0.001[c]
wuIs152 (sod-1 OE*)* [4]	71	31.0	31		
daf-16(mgDf50) [4]	74	15.6	15		
wuIs152 (sod-1 OE*) daf-16(mgDf50)* [4]	75	14.3	15	-0	0.022[c]
hsf-1(sy441) [1]	121	14.9	16		
wuIs152 (sod-1 OE*) hsf-1(sy441)* [1]	122	16.8	17	+6	<0.0001
hsf-1(sy441) [2]	68	15.3	16		
wuIs152 (sod-1 OE*) hsf-1(sy441)* [2]	71	17.3	18	+12	<0.0001
aak-2(ok524) [1]	119	15.9	15		
wuIs152 (sod-1 OE*) aak-2(ok524)* [1]	67	18.8	20	+33	<0.0001
aak-2(ok524) [2]	70	13.0	14		
wuIs152 (sod-1 OE*) aak-2(ok524)* [2]	67	19.1	18	+29	<0.0001
COMBINED DATA					
Trials [1] and [2]					
rol-6(dm)	189	18.6	18		
wuIs152(sod-1(+))	173	26.3	24	+33	<0.0001
aak-2(ok524)	189	14.9	15		
wuIs152 (sod-1 OE*) aak-2(ok524)*	134	19.2	20	+33	<0.0001
hsf-1(sy441)	189	15.1	16		
wuIs152 (sod-1 OE*) hsf-1(sy441)*	193	17.0	17	+6	<0.0001

[a] Calculated from median lifespan values

[b] Calculated by comparing to corresponding control condition using Wilcoxon test

[c] Calculated by comparing to *daf-16(mgDf50)* alone

All trials carried out at 20°C

Table S2: Additional survival data

Genotype [Trial number]	n	Mean Lifespan	Median Lifespan	% Change[a]	P-value[b]
rol-6(dm) [3]	79	17.9	18		
rol-6(dm) +NAC [3]	83	18.9	18		0.22[c]
wuIs152 (sod-1 OE*)* [3]	105	22.4	22		
wuIs152 +NAC [3]	111	24.6	24	+9	0.001[d]
rol-6(dm) [4]	92	19.3	18		
wuIs151 (ctl-1,2,3 OE*)* [4]	44	16.1	17	-6	<0.0001[c]
wuIs152 (sod-1 OE*)* [4]	78	25.6	25	+39	<0.0001[c]
wuIs154 (sod-1 OE*)* [4]	61	21.5	20	+11	0.0008[c]
wuIs151 (ctl-1,2,3 OE*) wuIs152 (sod-1* OE*)* [4]	87	24.3	23		0.21[d]
wuIs151 (ctl-1,2,3 OE*) wuIs154 (sod-1* OE*)* [4]	83	20.0	20		0.06[d]
Wild type [5]	43	18.0	18		
daf-16(mgDf50) [5]	48	16.9	18		0.34[c]
wuEx118 (sod-2 OE*)* [5]	62	20.1	21	+17	0.011[c]
wuIs155 (sod-2 OE*)* [5]	60	20.2	21	+17	0.0075[c]
wuIs156 (sod-2 OE*)* [5]	76	20.2	21	+17	0.017[c]
wuIs156 (sod-2 OE*) daf-16(mgDf50)* [5]	86	15.3	16	-11	<0.0001[e]
rol-6(dm) [6]	125	20.0	21		
wuIs151 (ctl-1,2,3 OE*)* [6]	237	20.7	21		0.31[c]
wuIs156 (sod-2 OE*)* [6]	130	23.9	25	+19	<.0001[c]
wuIs151 (ctl-1,2,3 OE*) wuIs156 (sod-2* OE*)* [6]	106	23.8	25		0.75[d]
rol-6(dm) [7]	93	16.9	15		
wuIs151 (ctl-1,2,3 OE*)* [7]	75	15.2	14	-7	0.0003[c]
wuIs156 (sod-2 OE*)* [7]	110	19.5	17	+13	0.0001[c]
wuIs151 (ctl-1,2,3 OE*) wuIs156 (sod-2* OE*)* [7]	202	20.8	21	+24	<.0001[d]
COMBINED DATA					
Trials [6] and [7]					
rol-6(dm) [6 + 7]	218	18.7	19		
wuIs151 (ctl-1,2,3 OE*)* [6 + 7]	312	19.4	19		0.42[c]
wuIs156 (sod-2 OE*)* [6 + 7]	240	21.9	21	+10	<.0001[c]
wuIs151 (ctl-1,2,3 OE*) wuIs156 (sod-2* OE*)* [6 + 7]	308	21.8	21		0.30[d]

Wild type [8]	121	21.8	20		
Wild type *ire-1(RNAi)* [8]	98	16.4	16	-20	<0.0001[c]
Wild type *xbp-1(RNAi)* [8]	131	19.3	18	-10	0.0003[c]
wuIs152 (sod-1 OE*)* [8]	54	32.6	36		
wuIs152 (sod-1 OE) *ire-1(RNAi)* [8]	91	21.5	21	-42	<0.0001[d]
wuIs152 (sod-1 OE) *xbp-1(RNAi)* [8]	109	27.8	28	-22	<0.0001[d]
Wild type [9]	127	19.7	19		
Wild type *ire-1(RNAi)* [9]	145	18.0	17		0.12[c]
Wild type *xbp-1(RNAi)* [9]	147	18.8	19		0.95[c]
wuIs152 (sod-1 OE*)* [9]	111	25.4	23		
wuIs152 (sod-1 OE) *ire-1(RNAi)* [9]	124	21.3	21	-9	0.0001
wuIs152 (sod-1 OE) *xbp-1(RNAi)* [9]	118	22.0	21	-9	0.0003[d]

[a] Calculated from median lifespan values
[b] Calculated by comparing to corresponding control condition using Wilcoxon test
[c] Compared to *rol-6(dm)* or wild type control
[d] Compared to corresponding SOD over-expressing strain
[e] Compared to *daf-16(mgDf50)* control

All trials carried out at 20°C
